# Elevated RIPK2 Expression in Atopic Dermatitis Skin Is Associated with Mixed Th1/Th2 Cytokine Responses in CD4^+^ T Cells

**DOI:** 10.3390/ijms27188253

**Published:** 2026-09-16

**Authors:** Raeda Mubariki, Ziad Khamaysi, Elie Kaabour, Mishlab Salih, Tal Belity, Elias Toubi, Eran Blacher, Zahava Vadasz

**Affiliations:** 1 Proteomic Unit, Bnai-Zion Medical Center, Haifa 3339419, Israel; 2 Faculty of Medicine, Technion-Israel Institute of Technology, Haifa 3525433, Israel; 3 Department of Dermatology, Rambam Medical Campus, The Rappaport Faculty of Medicine, Haifa 3525433, Israel; zkhamaysi@gmail.com (Z.K.);; 4 Department of Biological Chemistry, The Alexander Silberman Institute of Life Sciences, The Hebrew University of Jerusalem, Edmond J. Safra Campus Givat-Ram, Jerusalem 9190401, Israel; 5 The Center for Nanoscience and Nanotechnology, The Hebrew University of Jerusalem, Jerusalem 9190401, Israel; 6 Allergy and Clinical Immunology, The Holy Family Hospital, Nazareth 1600934, Israel

**Keywords:** atopic dermatitis, RIPK2, CD4 T cells, Th1/Th2 cytokines, immunopathogenesis

## Abstract

Receptor-interacting protein kinase 2 (RIPK2) is a critical mediator of the immune system leading to production of pro-inflammatory cytokines. Its role in the pathogenesis of atopic dermatitis (AD) has not been defined. This work aims to assess RIPK2 expression in the skin in AD and to evaluate the association with Th1-, Th2-, and Th17-related cytokine expression in lesional skin. AD patients’ skin biopsies and healthy controls were collected. An OPAL multiplex immunohistochemistry method was used to simultaneously detect TNF-α, IL-4, IL-13 and IL-17 in CD4^+^ RIPK2^+^ and CD4^+^ RIPK2^−^ T cells. Quantitative image analysis was performed, and group comparisons were made using non-parametric Mann–Whitney tests. Our results show that CD4^+^RIPK2^+^ cells were significantly abundant in AD skin, with mark increased expression of Th1, Th2, and Th17 cytokines simultaneously. However, CD4^+^ RIPK2^−^ T cells in AD overexpress TNFa without elevated Th2 cytokines, and IL-17^+^ cells were only marginally increased. In conclusion RIPK2 expression is upregulated in infiltrating CD4^+^ T cells in AD skin, with a mixed Th1/Th2 cytokine profile, suggesting that enhanced RIPK2 signaling contributes to the immune dysregulation in adult AD. These findings identify RIPK2 as a potential marker of Th1/Th2-high immune endotypes in AD and as a possible therapeutic target for intervention.

## 1. Introduction

Atopic dermatitis (AD) is a chronic inflammatory skin disease characterized by complex immune dysregulation, classically with a predominant Th2 immune skewing in acute lesions and a mixed Th1/Th2 profile in chronic disease [1]. Receptor-interacting protein kinase 2 (RIPK2) is an intracellular serine/threonine kinase that plays a central role in both innate and adaptive immune responses [2]. Upon sensing of peptidoglycan motifs by the intracellular pattern recognition receptors NOD1 and NOD2, RIPK2 is recruited to form a signaling complex that activates downstream NF-κB and MAPK pathways, leading to the production of pro-inflammatory cytokines and promotion of T-cell activation [3]. In immune cells, RIPK2 interacts not only with NOD receptors but also with other signaling receptors, such as certain Toll-like receptors and TNF receptor-associated factors, indicating additional functions outside of the canonical NOD pathways.

Recent studies have highlighted the involvement of RIPK2 in a variety of autoimmune and inflammatory disorders [2,4,5,6,7]. For example, increased RIPK2 expression has been observed in rheumatoid arthritis synovial tissues, and silencing RIPK2 in fibroblast-like synoviocytes reduced their inflammatory responses and proliferation [8]. Similarly, in models of inflammatory bowel disease, RIPK2-deficient mice were protected from developing colitis, underscoring the kinase’s contribution to Th1-mediated gut inflammation [5]. RIPK2 signaling also appears crucial for certain Th17 responses: joint inflammation in an experimental arthritis model was attenuated in RIPK2^−/−^ mice, which showed impaired IL-17 production [8]. On the Th2 side of immunity, RIPK2 has been implicated in allergic airway inflammation and asthma [4,6,7,9]. Animal studies demonstrate that RIPK2 promotes Th2 polarization; RIPK2 deficiency or inhibition led to reduced eosinophilic infiltration and lower levels of IL-4, IL-5, IL-13, and MCP-1 in ovalbumin-induced asthma models. Consistently, ongoing clinical trials are exploring RIPK2 inhibitors as potential treatments for Th2-driven diseases such as allergic asthma [10]. These collective findings position RIPK2 as a key modulator of both Th1 and Th2 immune pathways in various contexts.

Despite these insights, the role of RIPK2 in the pathogenesis of atopic dermatitis has not been investigated prior to this study. AD in adults, especially chronic disease, is known for a dynamic interplay of Th2 and Th1 immune responses, with elevated Th2 cytokines (e.g., IL-4, IL-13) driving acute exacerbations and Th1-associated cytokines (e.g., interferon-γ, TNF-α) contributing to chronic inflammation. A subset of patients also exhibit Th17/IL-23 pathway activation, particularly in certain ethnic populations (for instance, Asian AD phenotypes show combined Th2/Th17 polarization). This heterogeneity suggests distinct endotypes of AD, which could influence therapeutic responses to targeted treatments (such as Th2 cytokine inhibitors or JAK inhibitors) [11,12]. Given RIPK2’s broad role in modulating Th1 and Th2 responses, we hypothesized that RIPK2 may be upregulated in AD skin and associated with the skewed cytokine environment. In this study, we aimed to characterize RIPK2 expression in lesional skin of adults with moderate-to-severe AD, focusing on its expression in CD4^+^ T cells. We further investigated whether CD4^+^RIPK2^+^ T cells preferentially co-express Th1, Th2, or Th17 cytokines, in order to delineate how RIPK2 might relate to the immune profile (endotype) of AD. Elucidating the distribution of RIPK2-positive T cells in relation to cytokine expression may enhance our understanding of AD pathogenesis and inform more targeted therapeutic strategies for different patient subsets.

## 2. Results

### 2.1. RIPK2 Expression in AD Skin Biopsies

**Figure 1:**
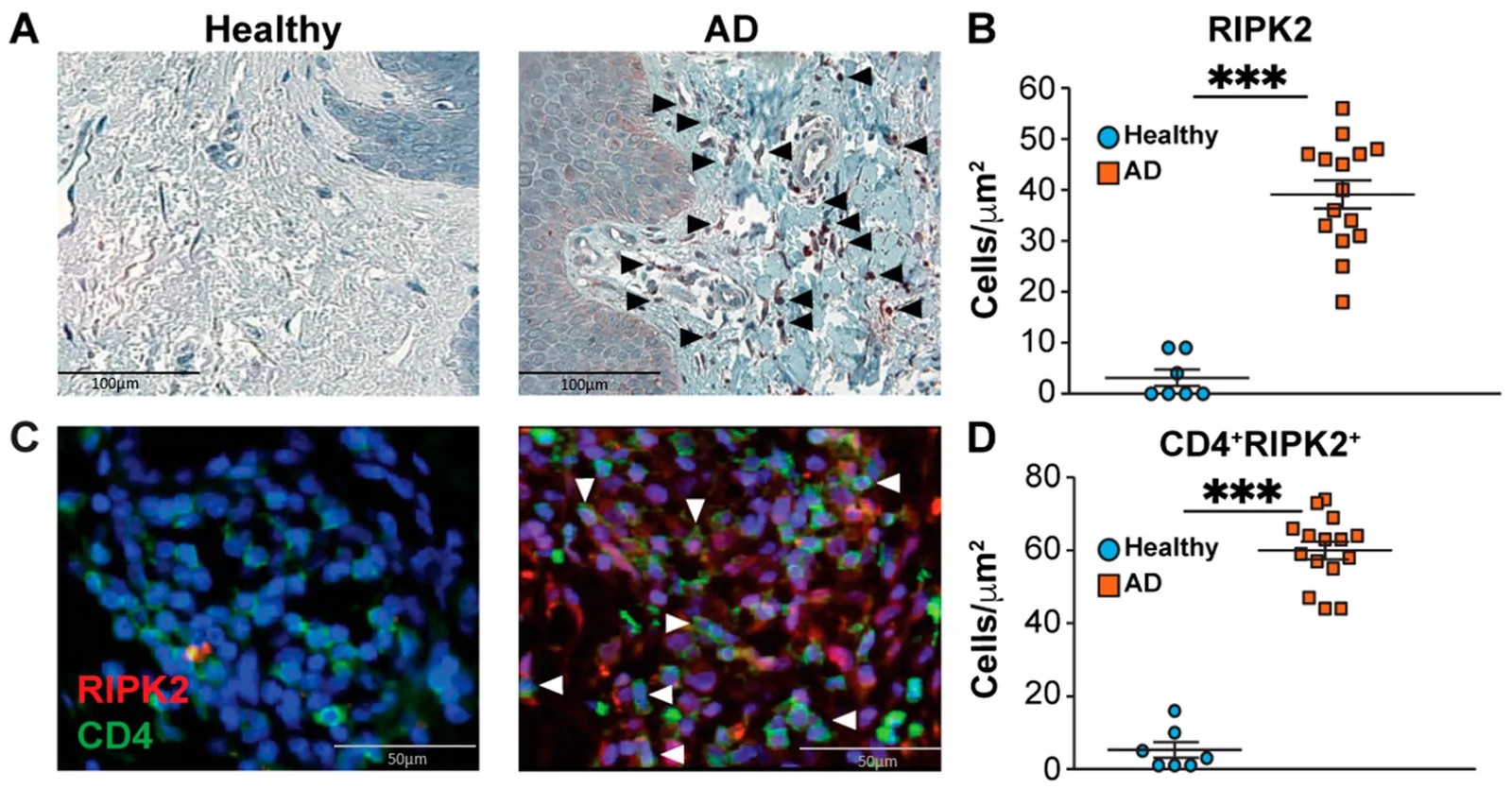
Increased RIPK2 expression in lesional atopic dermatitis and localization to CD4^+^ T cells. (**A**) Representative RIPK2 immunohistochemistry in lesional AD and healthy control skin (AEC, red-brown, hematoxylin counterstain). Scale bar = 100 µm. (**B**) Quantification of RIPK2^+^ cells (cells/µm^2^) in AD and controls (Mann–Whitney U test, mean ± SEM, U = 0, Z = −3.70, r = 0.79), *** *p* < 0.0001. (**C**) Dual immunofluorescence for CD4 (green) and RIPK2 (red) with DAPI nuclei (blue). Arrowheads indicate CD4^+^RIPK2^+^ cells. Scale bar = 50 µm. (**D**) Quantification of CD4^+^RIPK2^+^ cells (cells/µm^2^) in AD and controls (Mann–Whitney U test, mean ± SEM, U = 0, Z = −3.70, r = 0.79), *** *p* < 0.0001.

Given the multifaced immune regulatory roles played by RIPK2 in the context of inflammation, we first set out to assess RIPK2 expression in skin biopsies from AD patients and healthy controls (Table 1). We found that RIPK2 expression was substantially increased in lesional skin of AD patients compared to normal skin. On immunohistochemical staining, numerous AD skin cells exhibit strong cytoplasmic RIPK2 immunoreactivity, whereas normal skin shows only rare RIPK2-positive cells (Figure 1A). Quantitatively, AD biopsies showed more than a 12-fold higher density of RIPK2-positive cells relative to controls (39.1 ± 2.7 vs. 3.1 ± 1.6 RIPK2^+^cells per μm^2^, *p* < 0.0001) (Figure 1B). We next evaluated which cell types accounted for this increase. Using dual immunofluorescence, we observed that RIPK2 co-localized predominantly with CD4^+^ T cells in AD skin. In normal skin, very few CD4^+^RIPK2^+^ double-positive T cells are present, while AD skin contains many CD4^+^ cells that concurrently express RIPK2 (Figure 1C). Quantification of CD4^+^RIPK2^+^ cells demonstrated an 11-fold increase in AD compared to normal skin (mean 60.0 ± 2.4 vs. 5.3 ± 2.2 cells per μm^2^, *p* < 0.0001, Figure 1D). These data indicate that CD4^+^ T lymphocytes are a major source of elevated RIPK2 in AD lesions. 

### 2.2. Th1, Th2, and Th17 Cytokine Co-Expression in CD4^+^RIPK2^+^ T Cells

**Figure 2:**
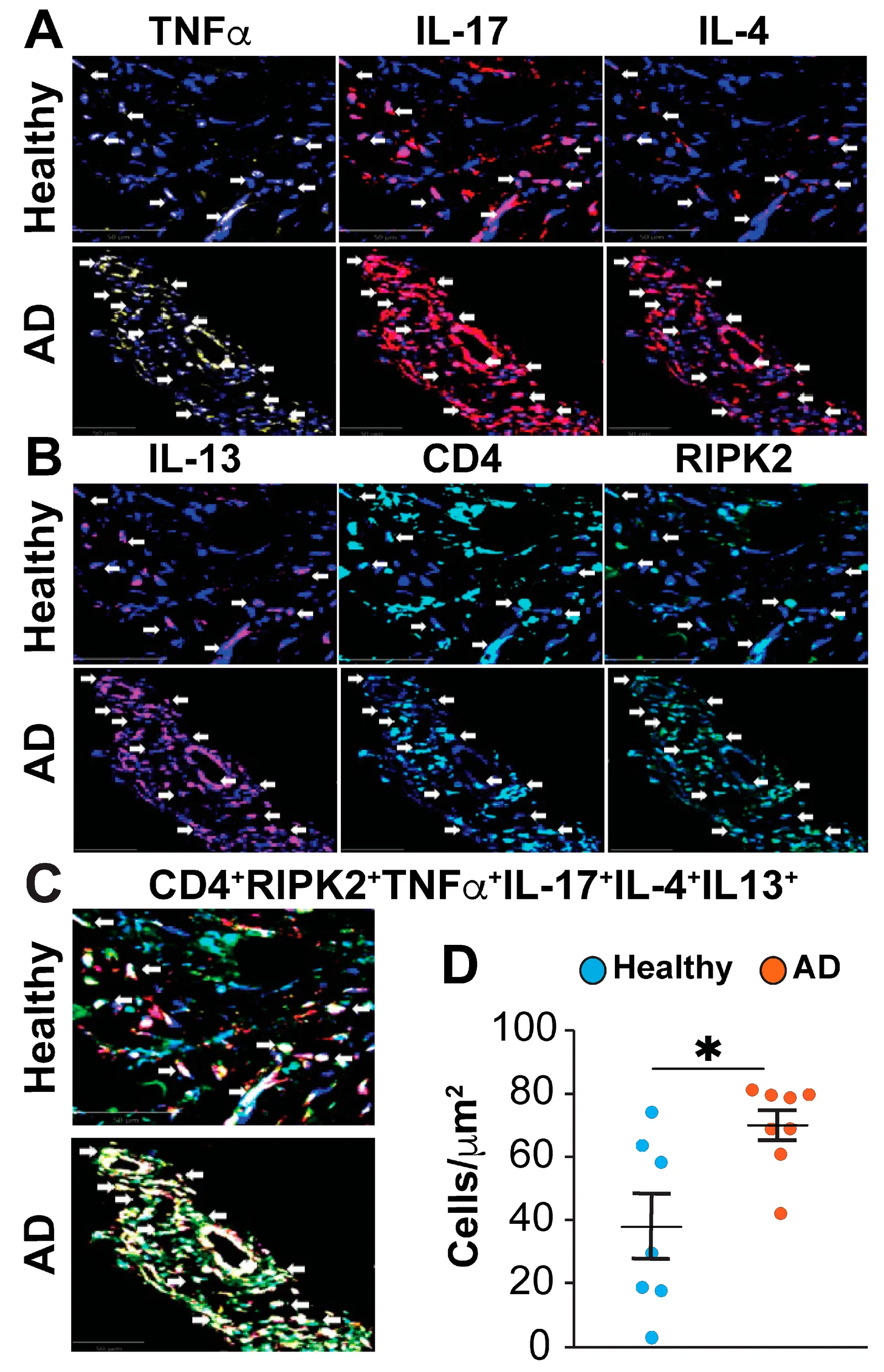
CD4^+^RIPK2^+^ T cells in lesional atopic dermatitis co-express Th1, Th2, and Th17 cytokines. Opal multiplex immunofluorescence for CD4 (cyan), RIPK2 (green), TNF-α (yellow), IL-17 (red), IL-4 (orange), and IL-13 (pink) in healthy control and lesional AD skin. (**A**) Representative fields showing TNF-α and IL-17 expression in CD4^+^RIPK2^+^ cells (control vs. AD). Scale bar = 50 µm. (**B**) Representative fields showing IL-4 and IL-13 expression in CD4^+^RIPK2^+^ cells (control vs. AD). Scale bar = 50 µm. (**C**) Higher magnification example of a CD4^+^RIPK2^+^ cell in AD co-expressing TNF-α, IL-17, IL-4, and IL-13 (arrows); matched control field shown for comparison. Scale bar = 20 µm. (**D**) Quantification of CD4^+^RIPK2^+^ cells co-expressing TNF-α, IL-17, IL-4, and IL-13 (cells/µm^2^) (Mann–Whitney U test; mean ± SEM, U = 7, Z = −2.43, r = 0.63), * *p* < 0.05.

To characterize the functional phenotype of the RIPK2-expressing T cells, we used multiplex immunohistochemistry to assess the co-expression of signature Th1, Th2, and Th17 cytokines in CD4^+^RIPK2^+^ cells. In healthy skin, CD4^+^RIPK2^+^ T cells were scarce and rarely co-expressed multiple cytokines (Figure 2A,B, upper panels). In contrast, AD skin contained a conspicuous population of CD4^+^RIPK2^+^ T cells that simultaneously expressed TNF-α (Th1), IL-17 (Th17), and the Th2 cytokines IL-4 and IL-13 (Figure 2A,B, lower panels). We observed a significant increase in the abundance of CD4^+^RIPK2^+^ T cells positive for all four cytokines by multiplex fluorescent staining from AD lesions (Figure 2C, upper panel. White arrows indicate the co-localization of these markers within the same cell). In the normal skin sample, by comparison, there are markedly fewer cells with any such multi-cytokine co-expression (Figure 2C, lower panel). Quantitative analysis revealed that the number of CD4^+^RIPK2^+^ T cells co-expressing all the measured cytokines was significantly higher in AD biopsies than in controls (Figure 2D). On average, AD skin had roughly 1.8 times more CD4^+^RIPK2^+^ T cells producing TNF-α, IL-17, IL-4, and IL-13 concurrently (70.0 ± 4.7 cells/μm^2^ in AD vs. 37.8 ± 10.3 in normal skin, *p* < 0.05). Taken together, these results show that RIPK2-positive T cells in AD tend to exhibit a mixed Th1/Th2/Th17 cytokine profile, whereas those in normal skin have a significantly lower cytokine expression profile. This suggests that RIPK2^+^ T cells in the AD microenvironment are functionally active and multi-cytokine-expressing CD4^+^RIPK2^+^ T cells, capable of producing multiple pro-inflammatory cytokines that contribute to the chronic inflammation.

### 2.3. Cytokine Expression in RIPK2-Negative CD4^+^ T Cells

**Figure 3:**
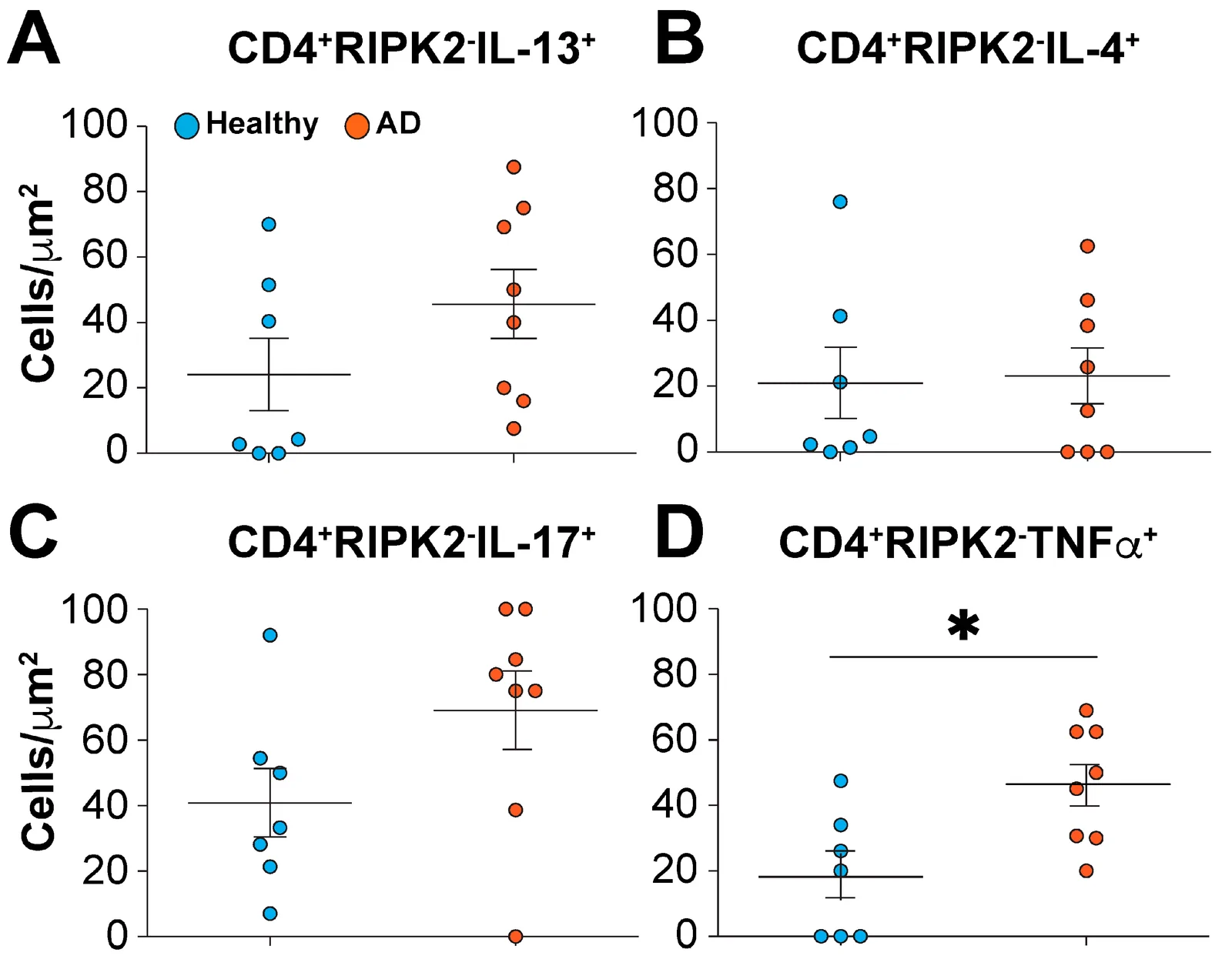
Cytokine expression in CD4^+^RIPK2^−^ T cells in lesional atopic dermatitis. Cytokine-positive CD4^+^RIPK2^−^ cells were quantified in lesional AD and healthy control skin. (**A**) IL-13 (U = 15, Z = −1.50, r = 0.39), (**B**) IL-4 (U = 27.50, Z = −0.06, r = 0.02), (**C**) IL-17 (U = 14, Z = −1.62, r = 0.42), and (**D**) TNF-α (U = 8.50, Z = −2.26, r = 0.58) (cells/µm^2^), * *p* < 0.05. TNF-α^+^ CD4^+^RIPK2^−^ cells are increased in AD, whereas IL-13, IL-4, and IL-17 are not significantly different between groups (Mann–Whitney U test, mean ± SEM).

For comparison, we examined cytokine expression in CD4^+^ T cells that lack RIPK2 (CD4^+^RIPK2^−^ cells) in AD versus healthy skin. Notably, the overall number of CD4^+^RIPK2^−^ T cells infiltrating AD skin was low relative to RIPK2-positive cells, reflecting the predominance of RIPK2 upregulation in AD T cells. Nonetheless, we evaluated the subset of RIPK2-negative T cells for Th1, Th2, and Th17 cytokines. In CD4^+^RIPK2^−^ cells, there was no significant difference in the frequency of IL-13^+^ cells between AD lesions and normal skin (Figure 3A), and similarly no difference in IL-4^+^ cells (Figure 3B), indicating that Th2 cytokine production was not elevated in T cells lacking RIPK2. The number of IL-17^+^ CD4^+^RIPK2^−^ T cells was slightly higher in AD samples (Figure 3C), but this trend did not reach statistical significance. In contrast, TNF-α-producing CD4^+^RIPK2^−^ T cells were significantly increased in AD skin compared to controls (Figure 3D). AD biopsies contained approximately twice as many CD4^+^RIPK2^−^ T cells expressing TNF-α as normal skin (46.2 ± 6.3 vs. 22.5 ± 8.5 cells/μm^2^, *p* < 0.05). While this significance was not preserved when performing family-wise Bonferroni correction (adjusted *p* = 0.1296), the large effect size (r = 0.58) confirms a substantial biological difference between the cohorts (Table 2). This finding suggests that even among T cells that do not express RIPK2, the AD environment favors a Th1-skewed response, as evidenced by elevated TNF-α production, while Th2 cytokines remain at baseline levels. It is also noteworthy that CD4^+^RIPK2^−^ cells constituted a minor fraction of total T cells in these AD lesions, implying that RIPK2 expression is a common feature of the T-cell infiltrate in moderate-to-severe AD.

## 3. Discussion

To our knowledge, this study is the first to implicate RIPK2 in the immunopathology of atopic dermatitis. We demonstrate that skin lesions from adults with moderate-to-severe AD have a markedly elevated presence of RIPK2, particularly within CD4^+^ T cells. These RIPK2-expressing T cells in AD skin are characterized by a mixed cytokine production profile, co-expressing key Th2 cytokines (IL-4, IL-13) as well as Th1 (TNF-α) and Th17 (IL-17) cytokines. This phenotype suggests that RIPK2 might be driving or marking a broad immune activation state in AD, bridging traditionally distinct T helper subsets. AD is increasingly recognized as a heterogeneous disease with multiple endotypes defined by underlying immune polarization (Th2-dominant, Th1/Th2-mixed, Th2/Th17-mixed, etc.) [11,12,13]. Our findings support the concept that adult chronic AD has a significant Th1 component superimposed on a Th2 background, and that RIPK2 may be a pivotal signaling molecule in this context. In line with this, previous observations have noted that long-standing or intrinsic AD lesions often exhibit upregulation of Th1-related cytokines (like interferon-γ and TNF-α) while still maintaining Th2-driven features such as high IgE levels. RIPK2 expression in T cells could be one factor that amplifies and sustains this combined Th1/Th2 response.

The mechanistic role of RIPK2 in T-cell activation provides a plausible explanation for our observations. RIPK2 is known to be required for optimal activation of NF-κB and MAPK pathways in T cells upon T-cell receptor and co-stimulatory signaling. Studies in RIPK2-deficient mice have shown impaired T-cell proliferation and cytokine production in response to stimulation. Moreover, RIPK2 deficiency confers resistance to certain T-cell-mediated inflammatory conditions, such as colitis and allograft rejection, underscoring its importance in pro-inflammatory T-cell functions. In our study, the prominent co-expression of TNF-α, IL-17, IL-4, and IL-13 in RIPK2-positive T cells suggests that RIPK2 signaling may enable T cells to become multi-cytokine-expressing CD4^+^RIPK2^+^ T cell effectors. One interesting finding was that IL-13 co-expression was significantly associated with RIPK2^+^ T cells in AD, whereas IL-4 co-expression was not as pronounced in those cells (despite IL-4 being a canonical Th2 cytokine). This could indicate a skewing of Th2 responses toward IL-13 in the chronic AD milieu, which is consistent with reports that IL-13 can be a dominant Th2 cytokine in chronic skin inflammation (and indeed IL-13 is a key target of newer biologics for AD). Meanwhile, CD4^+^RIPK2^−^ T cells in AD did not show increased IL-4 or IL-13, but did show an increase in TNF-α producers, reinforcing the idea that Th1 signals can operate independently of RIPK2 in some T cells, or that those cells might represent a different subset (e.g., tissue-resident Th1 cells) not reliant on RIPK2.

Our data carry potential clinical implications. First, the high level of RIPK2 in AD lesions (and particularly in cytokine-producing T cells) suggests that RIPK2 could serve as a biomarker for identifying an “inflamed T-cell” signature in AD. In practical terms, if one could measure RIPK2 expression in a skin biopsy or even in peripheral blood CD4^+^ T cells, it might help stratify patients by their immune activation status. For instance, we speculate that patients whose lesions harbor predominantly CD4^+^RIPK2^+^ T cells (with mixed Th1/Th2 cytokine production) would likely respond well to therapies targeting Th2 pathways (such as dupilumab or other IL-4/IL-13 axis inhibitors) because they clearly have an active Th2 component. They might also benefit from broader immunosuppressive agents like JAK inhibitors, which can simultaneously dampen Th1 and Th2 cytokine signaling. In fact, our findings align with the observed clinical efficacy of JAK1/2 inhibitors in AD, which reduce both Th2 and Th1 inflammation. Conversely, in patients whose skin inflammation is driven more by CD4^+^RIPK2^−^ T cells (which in our study were associated mainly with TNF-α and IL-17 production and not Th2 cytokines), one might predict a less robust response to Th2-targeted treatments. Such patients might require alternative strategies, for example, treatments targeting the Th1/Th17 axis (perhaps off-label use of TNF inhibitors, IL-17/IL-23 inhibitors, or other modalities not yet standard in AD). This personalized approach to AD treatment, guided by immune profiling, is an area of active interest, and our study adds RIPK2 as a novel candidate marker in this regard.

It is also worth noting that RIPK2 is an attractive therapeutic target in its own right. Small-molecule RIPK2 inhibitors are being developed and tested in inflammatory disease models. If RIPK2 is indeed a central node in the inflammatory network of AD, then RIPK2 inhibition could theoretically dampen multiple downstream cytokines (both Th1 and Th2), offering a broad anti-inflammatory effect. This approach might be beneficial for patients with mixed endotype disease. However, caution is warranted; RIPK2 also plays roles in host defense (especially in innate immune responses to microbial signals), so systemic inhibition could carry a risk of infections. Further research using ex vivo AD models or mouse models (if available for AD-like disease) would be needed to explore how RIPK2 blockade affects skin inflammation and barrier function.

Our study has some limitations. The sample size was relatively small, and all patients were from a single center in Israel; thus, the findings should be confirmed in larger and more ethnically diverse cohorts. Additionally, our approach was primarily descriptive and correlative: we showed associations between RIPK2 expression and cytokine profiles, but did not directly test causation. Future studies could involve functional experiments, for instance, isolating T cells from AD skin or blood and examining whether RIPK2 inhibition alters their cytokine production profile. Despite these limitations, our data clearly highlight a previously unrecognized aspect of AD immunopathology, namely, the involvement of RIPK2 in T-cell-mediated inflammation in the skin.

## 4. Materials and Methods

### 4.1. Study Population

Fifteen adult patients with chronic moderate-to-severe AD (10 males and 5 females, age range 23–77 years, mean age ~49) and seven healthy controls (4 males and 3 females, ages 32–79) were enrolled from the dermatology clinic at Rambam Health Care Campus starting November 2018. All AD patients met the Hanifin and Rajka diagnostic criteria for atopic dermatitis [12]. Patients had longstanding disease (at least 1 year duration) and active moderate-to-severe symptoms defined by: ≥10% body surface area (BSA) affected, Eczema Area and Severity Index (EASI) ≥12, Scoring Atopic Dermatitis (SCORAD) ≥20, and Investigator’s Global Assessment (IGA) score ≥3. Exclusion criteria included pregnancy or lactation and any serious comorbid conditions such as malignancy of skin (e.g., melanoma or T cell lymphoma of skin), active infection, or other inflammatory skin diseases (e.g., psoriasis). All AD patients were untreated systemically at the time of biopsy (aside from stable topical therapy or emollients); prior treatments—before the biopsy was taken—are summarized in Table 1 (which includes history of topical corticosteroids and calcineurin inhibitors in all patients, and various systemic therapies in some patients, such as cyclosporine, methotrexate, azathioprine, plasmapheresis, mycophenolate, and dupilumab). The study was approved by the Rambam Health Campus ethics committee (approval no. RMB-0242-22, approval date on 6 July 2022) and conducted in accordance with the Declaration of Helsinki. The whole study was exempt from informed consent due to retrospective unidentified samples. 

### 4.2. Skin Immunohistochemistry (IHC)

Formalin-fixed, paraffin-embedded skin biopsy specimens from lesional AD skin (*n* = 15) and normal skin (*n* = 7) were sectioned at 5 μm thickness. IHC staining for RIPK2 was performed on deparaffinized tissue sections. After rehydration through xylene and graded ethanols, endogenous peroxidase was quenched with 1% H_2_O_2_ in methanol for 30 min. Antigen retrieval was carried out in an EDTA buffer (2 mM, pH 8.0) under pressure cooker conditions. Sections were blocked with 1% bovine serum albumin (BSA) for 1 h at room temperature, and then incubated overnight at 4 °C with primary antibody against human RIPK2 (rabbit polyclonal, 1:500 dilution, Abcam Ltd., Cambridge, UK). The following day, slides were washed in phosphate-buffered saline (PBS) and incubated for 1 h at room temperature with a secondary detection reagent (HISTOFINE Simple Stain Max PO multi-link HRP polymer, Nichirei Biosciences, Tokyo, Japan). Color development was achieved using an AEC chromogen substrate (Nichirei Biosciences) for 17 min, yielding a red-brown reaction product at sites of RIPK2 expression. Finally, sections were counterstained with hematoxylin, rinsed, dehydrated, and mounted with permanent medium. Negative control slides (omitting the primary antibody) were included to confirm staining specificity. Photomicrographs of stained sections were captured using an Olympus DP70 digital camera attached to a light microscope (Olympus Corporation, Tokyo, Japan). Quantitative analysis of RIPK2-positive cells was performed by counting positively stained cells in five high-power fields per sample (400× magnification) and normalizing to tissue area (cells per μm^2^) using ImageJ software (v1.54p, NIH, Bethesda, MD, USA).

### 4.3. Skin Immunofluorescence (IF)

Adjacent 5 μm tissue sections from the same biopsies were used for dual immunofluorescence to identify CD4^+^ T cells co-expressing RIPK2. After deparaffinization and rehydration, heat-induced antigen retrieval was performed in EDTA buffer (pH 8.0) as above. Non-specific binding was blocked with 3% glycine for 1 h followed by 1% BSA for an additional hour. Slides were then incubated overnight at 4 °C with a combination of two primary antibodies: rabbit anti-human RIPK2 (1:1000, Abcam Ltd., Cambridge, UK) and mouse anti-human CD4 (1:25, Thermo Fisher Scientific, Waltham, MA, USA). On the next day, after thorough PBS washes, slides were incubated with species-appropriate fluorescent secondary antibodies for 45 min at room temperature (e.g., Alexa Fluor 488 anti-mouse IgG for CD4 and Alexa Fluor 594 anti-rabbit IgG for RIPK2, or equivalent, to yield distinct green and red signals). Nuclei were counterstained with DAPI. Negative control slides (omitting the primary antibody) were included to confirm staining specificity. Slides were mounted in fluorescence mounting medium and scanned using an automated slide scanner. Digital fluorescence images were analyzed with ImageJ (v1.54p, NIH, Bethesda, MD, USA) to count CD4^+^RIPK2^+^ double-positive cells. For each sample, multiple fields were examined to ensure representation of the entire tissue section.

### 4.4. OPAL Multiplexed Immunohistochemistry

To simultaneously assess co-expression of multiple cytokines in CD4^+^RIPK2^+^ cells, we employed an OPAL multiplex IHC protocol (Akoya Biosciences, Inc., Marlborough, MA, USA) on a subset of the samples (AD *n* = 8, controls *n* = 7). Paraffin sections (5 μm) were stained using iterative cycles of tyramide signal amplification. In brief, after dewaxing, rehydration, and fixation, sequential antigen retrieval steps were performed (alternating between EDTA buffer pH 8.0 and Citrate buffer pH 6.0 for different targets). The following primary antibodies were applied in successive rounds: rabbit anti-RIPK2 (1:100, Abcam Ltd., Cambridge, UK)), rabbit anti-CD4 (1:300, Abcam Ltd., Cambridge, UK)), rabbit anti-IL-17 (1:200, Abcam Ltd., Cambridge, UK)), rabbit anti-IL-13 (1:500, Abcam Ltd, Cambridge, UK)), rabbit monoclonal anti-IL-4 (1:100, Abcam Ltd., Cambridge, UK)), and rabbit monoclonal anti-TNF-α (1:100, Abcam Ltd., Cambridge, UK)). Each primary incubation was for 1 h at room temperature (or overnight at 4 °C), followed by secondary HRP polymer application and then an Opal fluorophore-conjugated tyramide substrate incubation (each Opal reagent specific to a given marker, yielding a distinct fluorescent wavelength). After each staining round, slides were microwave-treated to strip antibodies while leaving the covalently deposited fluorescent signal, allowing the next marker to be stained sequentially. This multiplex staining resulted in all six markers (CD4, RIPK2, IL-17, IL-13, IL-4, TNF-α) being labeled on the same tissue section with minimal spectral overlap. All antibodies were evaluated prior to OPAL multiplex IHC utilizing a regular immunofluorescence staining protocol as follows: 1. For the antigen retrieval buffer used, if not specified by the manufacturer, one slide was boiled with EDTA buffer and another with Citrate buffer; primary and secondary antibodies were added and slides were evaluated under a fluorescent microscope to determine in which buffer the best signal was detected. 2. For primary antibody dilution, each antibody was evaluated using two different dilutions depending on the manufacturer’s recommendation or published papers; secondary antibodies were added and slides were evaluated under a fluorescent microscope to determine fluorescence detection. 3. Negative control slides (omitting the primary antibody) were included to confirm staining specificity. Multispectral images were acquired using the Akoya PhenoCycler Fusion Imaging System with PhenoCycler Fusion software 2.2.0 and images were analyzed with QuPath (version 0.5.1, University of Edinburgh, Edinburgh, UK) or similar image analysis software. We specifically quantified the number of CD4^+^RIPK2^+^ T cells that co-expressed each of the cytokines of interest. Similarly, for comparison, we quantified cytokine-positive cells among CD4^+^RIPK2^−^ T cells in the same specimens.

### 4.5. Statistical Analysis

All quantitative data are presented as mean ± standard error (SEM). Comparisons between AD and control groups were made using the non-parametric Mann–Whitney U test (given the relatively small sample size and non-normal distribution of counts). A two-tailed *p* value ≤ 0.05 was considered statistically significant. Data analysis was performed using GraphPad Prism (version 8.0.2.263, Boston, MA, USA). 

For OPAL multiplexed immunohistochemistry analysis, Z-scores were calculated manually using the following formula: Z = $\frac{U-\mu_{U}}{\sigma_{U}}$, where $\frac{n_{1}n_{2}}{2}$, and $\sigma_{U}$ = $\sqrt{\frac{n_{1}n_{2}(n_{1}+n_{2}+1)}{12}}$.

The effect size (r) was quantified as follows: r = $\frac{\left|Z\right|}{\sqrt{N}}$ where N is the total sample size across both cohorts (n_1_ + n_2_). Effect size strengths were characterised based on standard Cohen’s criteria as follows: 0.10 ≤ r ≤ 0.30 small, 0.30 ≤ r ≤ 0.50 medium, and r ≥ 0.50 large. Bonferroni correction of significance was calculated as: P_adj_ = P_raw_ × 6. 

## 5. Conclusions

In summary, we report that RIPK2 is highly upregulated in the skin of adult patients with moderate-to-severe atopic dermatitis and is chiefly expressed in infiltrating CD4^+^ T cells. These RIPK2^+^ T cells in AD are associated with a combined Th1 and Th2 cytokine response, reflecting the chronic inflammatory milieu of the disease. In contrast, T cells lacking RIPK2 show a more restricted cytokine profile. These differences in RIPK2 expression and cytokine secretion could be due to potentially different AD endogroups, and different stages of chronicity. Our preliminary findings suggest that RIPK2 may serve as a key regulator of the immune network in chronic AD, potentially driving the concurrent Th1/Th2 activation. Targeting RIPK2, or using it as a biomarker to stratify patients by immune endotype, could represent a novel avenue for improving the management of atopic dermatitis; this could be a potentially valuable tool for assessing the treatment effect of therapies such as JAK inhibitors or monoclonal antibodies. The limitation of this study is the small number of studied patients, mainly those of the RIPK2^−^ group. Ultimately, this work lays the foundation for future studies to further elucidate RIPK2’s role and to explore RIPK2-directed therapies in the context of AD.

## Figures and Tables

**Table 1:** Patients’ characteristics.

Participants’ Characteristics (*n* = 15)	
Sex	Female = 5, Male = 10
Age	23–77 years
	(mean = ±49 years)
IgE	Normal (5/15)
	Mild (2/15)
	High (8/15)
Comorbidities	Allergic Rhinitis
	Asthma
	Hyperlipidemia
	Obesity
	Vitiligo
	Hypertension
	Diabetes
Previous Treatment-Topical	Steroids (15/15)
	Skin oil (15/15)
	Anti-histamines (15/15)
	Phototherapy (10/15)
Previous Treatment-Systemic	Steroids (13/15)
	Cyclosporine (2/15)
	Methotrexate (7/15)
	Imuran (6/15)
	Plasmapheresis (1/15)
	Cellcept (1/15)
	Dupixent (4/15)

**Table 2:** Statistical plus multiple comparison correction (k = 6).

	U Value	Z Score	*P*_value_	*P*_adj_	Effect Size (r)	Effect Size Strength
CD4^+^RIPK2^+^	0.00	−3.70	<0.0001	<0.0006	0.79	Large
RIPK2^+^TNF-α^+^IL-17^+^IL-4^+^IL-13^+^	7.00	−2.43	0.0140	0.0840	0.63	Large
CD4^+^RIPK2^-^TNF-α^+^	8.50	−2.26	0.0216	0.1296	0.58	Large
CD4^+^RIPK2^-^IL-17^+^	14.00	−1.62	0.1125	0.6750	0.42	Medium
CD4^+^RIPK2^-^IL-13^+^	15.00	−1.50	0.1453	0.8718	0.39	Medium

## Data Availability

The original contributions presented in this study are included in the article. Further inquiries can be directed to the corresponding author.

## References

[1] Boothe W.D., Tarbox J.A., Tarbox M.B. (2024). Atopic Dermatitis: Pathophysiology. Adv. Exp. Med. Biol..

[2] Hofmann S.R., Girschick L., Stein R., Schulze F. (2021). Immune-modulating effects of receptor interacting protein 2 (RIP2) in autoinflammation and immunity. Clin. Immunol..

[3] Magalhaes J.G., Lee J., Geddes K., Rubino S., Philpott D.J., Girardin S.E. (2011). Essential role of Rip2 in the modulation of innate and adaptive immunity triggered by Nod1 and Nod2 ligands. Eur. J. Immunol..

[4] Goh F.Y., Cook K.L., Upton N., Tao L., Lah L.C., Leung B.P., Wong W.F. (2013). Receptor-interacting protein 2 gene silencing attenuates allergic airway inflammation. J. Immunol..

[5] Honjo H., Watanabe T., Kamata K., Minaga K., Kudo M. (2021). RIPK2 as a New Therapeutic Target in Inflammatory Bowel Diseases. Front. Pharmacol..

[6] Miller M.H., Shehat M.G., Alcedo K.P., Spinel L.P., Soulakova J., Tigno-Aranjuez J.T. (2018). Frontline Science: RIP2 promotes house dust mite-induced allergic airway inflammation. J. Leukoc. Biol..

[7] Miller M.H., Shehat M.G., Tigno-Aranjuez J.T. (2020). Immune Modulation of Allergic Asthma by Early Pharmacological Inhibition of RIP2. Immunohorizons.

[8] Vieira S.M., Cunha T.M., França R.F., Pinto L.G., Talbot J., Turato W.M., Lemos H.P., Lima J.B., Verri W.A., Almeida S.C. (2012). Joint NOD2/RIPK2 signaling regulates IL-17 axis and contributes to the development of experimental arthritis. J. Immunol..

[9] Alvarez-Simon D., Ait Yahia S., Audousset C., Fanton d’Andon M., Chamaillard M., Gomperts Boneca I., Tsicopoulos A. (2024). Local receptor-interacting protein kinase 2 inhibition mitigates house dust mite-induced asthma. Eur. Respir. J..

[10] Pham A.-T., Ghilardi A.F., Sun L. (2023). Recent advances in the development of RIPK2 modulators for the treatment of inflammatory diseases. Front. Pharmacol..

[11] Fyhrquist N., Yang Y., Karisola P., Alenius H. (2025). Endotypes of atopic dermatitis. J. Allergy Clin. Immunol..

[12] Wollenberg A., Barbarot S., Bieber T., Christen-Zaech S., Deleuran M., Fink-Wagner A., Gieler U., Girolomoni G., Lau S., Muraro A. (2018). Consensus-based European guidelines for treatment of atopic eczema (atopic dermatitis) in adults and children: Part I. J. Eur. Acad. Dermatol. Venereol..

[13] Slominski R.M., Raman C., Jetten A.M., Slominski A.T. (2025). Neuro–immuno–endocrinology of the skin: How environment regulates body homeostasis. Nat. Rev. Endocrinol..

